# The effects of virtual logotherapy on health-promoting lifestyle among single-parent adolescent girls during the coronavirus disease 2019 pandemic: a randomized clinical trial

**DOI:** 10.1186/s12905-023-02431-y

**Published:** 2023-05-19

**Authors:** Fatemeh Hosseinzadeh, Reza Negarandeh, Akram Sadat Sadat-Hoseini, Shahzad Pashaeypoor

**Affiliations:** 1 Department of Community Health Nursing, School of Nursing and Midwifery, Tehran University of Medical Sciences, Tehran, Iran; 2 Nursing and Midwifery Care Research Center, School of Nursing and Midwifery, Tehran University of Medical Sciences, Tehran, Iran; 3 Department of Pediatrics and Intensive Care of Neonatal, School of Nursing and Midwifery, Research Center of Quran, Hadith and Medicine, Tehran University of Medical Sciences, Tehran, Iran; 4 Department of Community Health and Geriatric Nursing, School of Nursing and Midwifery, Tehran University of Medical Sciences, Tehran, Iran; 5 Community-Based Participatory Research Center, Iranian Institute for Reduction of High–Risk Behaviors, Tehran University of Medical Sciences, Tehran, Iran

**Keywords:** Logotherapy, Health-promoting lifestyle, Health, Single-parent adolescents

## Abstract

Single-parent adolescents are vulnerable individuals and it is necessary to improve their health, particularly during epidemics. This study aimed to investigate the effects of virtual logotherapy (VL) on health-promoting lifestyle (HPL) among single-parent adolescent girls during the COVID-19 pandemic. This single-blind randomized clinical trial was conducted on 88 single-parent adolescent girls recruited from the support organization for vulnerable individuals in Tehran, Iran. They were randomly allocated to a control and an intervention group through block randomization. Participants in the intervention group received VL in three–five person groups in 90 min biweekly sessions. The Adolescent Health Promotion Short-Form was used to assess HPL. Data were analyzed using the SPSS software (v. 26.0) and through the independent-sample *t*, Chi-square, Fisher’s exact, and Mann–Whitney *U* tests. There was no significant difference between the intervention and the control groups respecting the pretest mean score of HPL (73.58±16.74 vs. 72.80±9.30; *P*=0.085). However, the posttest mean score of HPL in the intervention group (82 with an interquartile range of 78–90) was significantly more than the control group (71.50 with an interquartile range of 63.25–84.50) (*P*=0.001). Moreover, after adjusting the effects of the significant between-group differences respecting pretest mean scores, the pretest–posttest differences of the mean scores of HPL and all its dimensions in the intervention group were significantly more than the control group (*P*<0.05). VL is effective in significantly improving HPL among single-parent adolescent girls. Healthcare authorities are recommended to use VL for health promotion among single-parent adolescents.

**Trial registration**

This research was registered (17/05/2020) in the www.thaiclinicaltrials.org with registration number: TCTR20200517001.

## Introduction

Health-promoting lifestyle is a multidimensional model of perceptions and activities that begin with personal motivation and help to improve and promote health and self-care [1]. Health-promoting lifestyle (HPL) is a main predictor of physical and mental health [2] and shows the human desire for excellence [1]. It refers to actions with positive effects on health [3] and consists of six main dimensions, namely nutrition, physical activity, life appreciation, social support, health responsibility, and stress management [4]. Statistics show that 53% of all deaths are related to lifestyle [5]. Moreover, most chronic and non-communicable diseases such as obesity, cardiovascular disease, cancer, and diabetes mellitus are due to modifiable lifestyle-related risk factors such as tobacco smoking, alcohol consumption, unhealthy eating, and immobility [3, 5].

The culture that dominates the society is one of the factors that can affect people's behavior and lifestyle [6] and lifestyle in Iran is influenced by religion and Iranian culture and due to the wide cultural diversity in Iran, various lifestyles that affect people's behavior and health [7].

Lifestyle and HPL usually develop during adolescence [4]. Adolescence is considered as one of the most important stages of life in any society because the health of adolescents is an important foundation for the health of society [8]. Changes during adolescence affect lifestyle behaviors such as eating, sleeping, physical activity, and weight control [9]. Behaviors and habits developed during adolescence can extend to adulthood and affect health in later stages of life. Therefore, it is necessary to pay careful attention to adolescents’ lifestyle behaviors [4, 9, 10].

Adolescents are at risk for many different high-risk behaviors and health problems such as immobility, unhealthy eating, tobacco smoking, unprotected sexual relationships, mental disorders, violence, and suicide which can seriously threaten their adulthood health [8, 11, 12]. Statistics show that one tenth of 13–15 year-old adolescents smoke tobacco, one seventh of 10–19 year-old adolescents suffer from mental disorders, and 42% of male adolescents and 37% of female adolescents are at risk for violent behaviors [12]. In single-parent families, adolescents experience more frequent and more complex problems. Single-parent families are families with a single parent due to divorce, death of a parent, extramarital pregnancy, or adoption [13, 14]. In these families, adolescents have limited parental support and parents may pay lower attention to their children’s health-related needs [15]. Moreover, children in these families, particularly girls, experience more problems respecting academic achievement, self-confidence, social status, and interpersonal relationships and have more physical, mental, behavioral, and social disorders [16–18]. Moreover, these adolescents are disappointed about their future and most of them experience financial problems as well as problems in communication with their parents [16]. The most important reasons for the higher prevalence of these problems in single-parent families are limited financial resources, limited investment in health, lower parental supervision, and more financial and social problems [15, 18].

Meaning-based approaches and logotherapy are among the strategies with potentially positive effects on adolescents’ problems [19]. Logotherapy is a type of active guidance therapy developed by Victor Frankl to help individuals during difficult and critical conditions of life. Logotherapy holds that the bases of meaninglessness in life are ignorance, frustration, and despair and states that individuals will no longer feel frustration and despair when they find the latent meaning in their life [20]. Logotherapy attempts to make individuals aware of their responsibilities and remind them of the fact that their life is the result of their choices and their future is formed based on their current decisions [21].

Different studies have so far been conducted into the effects of logotherapy on the different aspects of health. For example, a study showed that participation in logotherapy-related programs had a negative relationship with suicidal thoughts and depression symptoms and positive relationship with self-esteem and perceived social support [22]. Another study reported that meaningfulness in life had a significant negative relationship with depression and anxiety and significant positive relationship with hopefulness and physical, emotional, functional, and social well-being [22].

Logotherapy needs therapist-client interaction [22]. However, the coronavirus disease 2019 (COVID-19) pandemic had a broad range impact on people's physical and mental health, lifestyle and meaning in life [23] and has affected all health-related measures [24, 25]. Restrictions imposed due to the pandemic, such as social distancing, have created a great need for telehealth methods in order to ensure the access of all healthcare clients to healthcare services [26–28]. Evidence also shows the increasing use of virtual methods by healthcare providers [24]. Compared with traditional methods, the benefits of virtual methods are lower a risk of COVID-19 transmission, greater care continuity, improved care efficiency, faster access to healthcare services, more effective communication between healthcare providers and clients, and more flexibility in care provision. However, virtual care has some challenges such as clients’ preference for face-to-face services, limited access of some individuals to information technology, the inappropriateness of virtual methods for some types of healthcare services, lack of official guidelines, heavier workload, greater need for financial resources, poor organizational culture for virtual care, technical and practical problems, and limited supervision [29].

Different studies used logotherapy to improve the different aspects of health. For instance, a study showed that a meaning-based intervention by nurses during the COVID-19 pandemic significantly reduced stress and depression and improved meaningfulness among college students, which could lead to reduced psychological distress and improved mental health [27]. Another study showed that logotherapy significantly improved social relationships and reduced the sense of loneliness [30]. Similarly, a study on the effectiveness of group logotherapy on the psychological well-being and happiness of students, found that logotherapy improved autonomy, environmental mastery, personal growth, positive relationships, purposefulness in life, self-acceptance, and happiness among orphan students and students with irresponsible parents [31]. Other studies also highlighted that logotherapy can be used to reduce anxiety and depression and improve quality of life, social functioning, hopefulness, meaningfulness, and sense of responsibility [32–38]. A systematic review also recommended web-based logotherapy as a good option to reduce perceived isolation and improve welfare among students during the COVID-19 pandemic [39].

Despite the wealth of studies into the effects of logotherapy on the different aspects of health and life, there are limited data about the effects of virtual logotherapy (VL) on HPL, particularly during epidemics such as the current COVID-19 pandemic. Therefore, the present study was conducted to narrow this gap. The study aimed to investigate the effects of VL on HPL among single-parent adolescent girls during the COVID-19 pandemic.

## Methods

### Design

This single-blind randomized clinical trial was conducted in Iran.

### Participants and setting

Study setting was the support organization for vulnerable individuals in Tehran, Iran, and study population consisted of all single-parent adolescent girls who referred to the study setting. Participants were 88 girls who were willing to participate in the study and met the following eligibility criteria: age 13–18 years, having just one parent, and no self-report history of psychological disorders or specific diseases. More than two absences from the intervention sessions were the exclusion criterion. It should be noted that ethical approval was given by the organizational ethics committee of the nursing-midwifery and rehabilitation faculty and participants entered the study voluntarily and completed the informed consent form. Participants were randomly allocated to a control and an intervention group through block randomization and using an online randomization module (www.randomization.com). The allocation sequence was concealed using 88 cards in 88 opaque envelopes. One envelope was randomly opened for each new participant and she was allocated to either of the groups based on the envelope card.

**Fig. 1:**
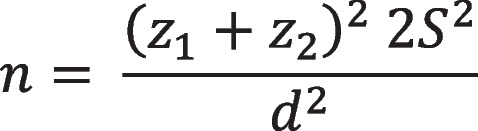
Sample size calculation formula

Sample size was calculated using the results of a study into the effects of education for student health ambassadors on HPL among adolescent girls. Accordingly, with a confidence level of 0.85, a power of 0.80, a standard deviation of 15 for the HPL score, and at least 10 score increase in the mean score of HPL after VL to be considered significant [40], sample size was determined to be 35 per group (Fig. 1). Nonetheless, the sample size was increased to 44 based on a probable attrition rate of 20%.

### Instruments

Data were collected using a demographic questionnaire and the Adolescent Health Promotion Short-Form. The demographic questionnaire had ten items on age, educational year, weight, height, family size, birth rank, parent (father or mother), adequacy of monthly family income, and parent’s educational level and occupation.

The Adolescent Health Promotion Short-Form was used for HPL assessment. This scale has 21 items in the following six main dimensions: nutrition (*n* = three, items one–three), social support (*n* = four, items four–seven), health responsibility (*n* = four, items eight–11), life appreciation (*n* = four, items 12–15), physical activity (*n* = three, items 16–18), and stress management (*n* = three, items 19–21). Items are scored on a five-point scale from one (“Never”) to five (“Always”) and the possible total score of the scale is 21–105, with higher scores showing healthier HPL. Chen et al., the developers of the scale, confirmed its acceptable construct, convergent, and discriminant validity through exploratory and confirmatory factor analysis and confirmed its reliability with a Cronbach’s alpha of 0.905 [41]. Another study also confirmed the acceptable face and content validity of the Persian version of the scale and its acceptable reliability with a test–retest intraclass correlation coefficient of 0.8 and a Cronbach’s alpha of 0.83 [40].

Participants in the intervention group received VL in three–five person small groups in eight 90-min biweekly online and offline sessions. The participants resided in their respective houses and used their mobile phones and the intervention was carried out using the live broadcasting of educational software of the Ministry of Education and voice calls in WhatsApp messenger, as well as the possibility of sending files in this software.

Educational materials were provided through lectures and group discussions. Moreover, real stories of famous people with enormous success despite disability or problems such as parent loss were narrated to participants and discussed. The important points of each session were also provided to participants through pamphlets and pictures for the purpose of offline use. The audio file of each session was also provided to them at the end of the session. The VL program (Table 1) was developed based on Breitbart and Applebaum’s studies [42]. In order to check its content validity, it was given to five professors of the Faculty of Nursing and Midwifery of Tehran University of Medical Sciences, and its content validity was confirmed (Cronbach’s alpha = 0.92). The intervention consisted of eight sessions and in these sessions, group discussion and Socratic teaching technique were used. The topics discussed in these meetings included meaningfulness of life, the meaning therapy approach and its dimensions, sources and methods of acquiring meaning in life. In addition, during the sessions, the researcher tried to encourage the participants to search for a specific meaning in their lives. More details are given in Table 1. Also the participants in both groups completed the Adolescent Health Promotion Short-Form before and eight weeks after the study intervention through WhatsApp.

**Table 1:** The content of the logotherapy sessions

Session	
1	Introduction; organization of the sessions; acquaintance of group members with each other; determination of group rules and norms; explanation about healthy lifestyle and logotherapy
2	Encouragement of group members to share their experiences; lecture about life meaningfulness; narration of stories of famous people who achieved enormous success despite disability or problems
3	Education about logotherapy (responsibility, anxiety, meaning seeking, suffering, and self-actualization); encouragement of members to express their concerns; discussion of the role of other individuals and life problems in giving meaning to life. The Socratic method of teaching was used in this session
4	Improvement of self-awareness of cognitions related to violent behaviors and despair; explanation about presence at the present moment
5	Introduction and analysis of the hierarchy of needs; education about the methods for meaning seeking during work, love, and suffering through group discussion; encouragement of group members to share their ideas about love and suffering
6	Discussion about responsibility; encouragement of group members to accept responsibility towards self, others, and life; group members’ introduction of successful and responsible individuals
7	Understanding and accepting loneliness as an inevitable reality; understanding the non-opposition of loneliness with closeness to others; understanding the role of intimacy in coping with loneliness; analysis of the sentence “He who has a why to live for can bear almost any how”
8	Termination Session: Understanding the concept of self-actualization and self-transcendence; characteristics of self-actualization; development of transcendence among group members through reducing their disappointing behaviors; summarizing what was said in the previous sessions

### Data analysis

Data were analyzed using the SPSS software (v. 26.0). Pretest and posttest data were described using the measures of descriptive statistics (namely mean, median, standard deviation, and interquartile range) and analyzed using the independent-sample *t*, Chi-square, Fisher’s exact, and Mann–Whitney *U* tests.

## Results

**Fig. 2:**
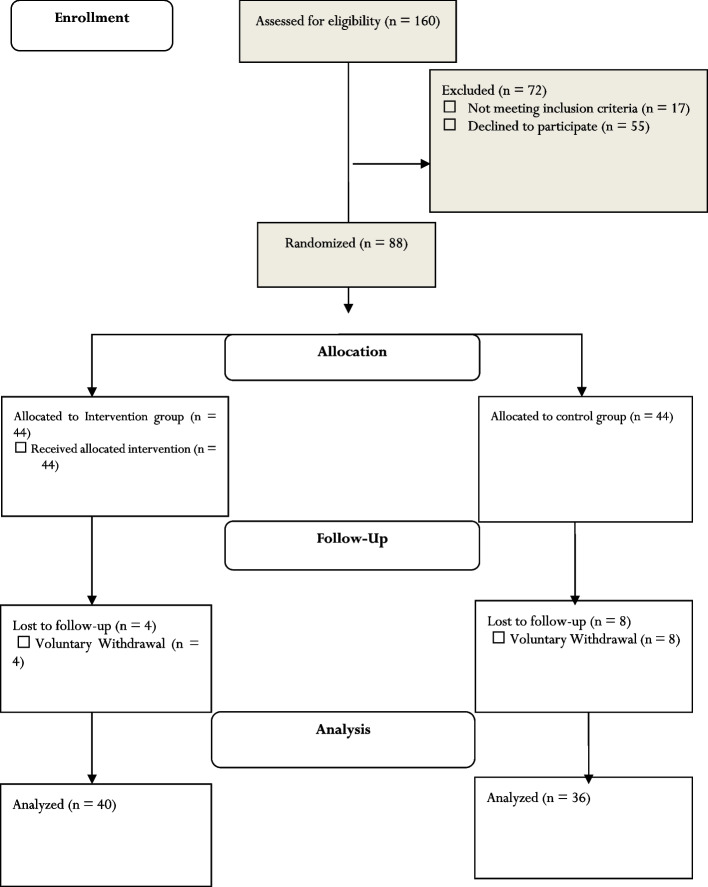
The flow diagram of the study

Initially, 88 eligible girls were recruited to the study. Eight participants from the control group were excluded due to loss to follow-up at posttest and four participants from the intervention group were excluded due to voluntary withdrawal (Fig. 2).

The means of participants’ age, height, weight, and family size in the intervention and the control groups were 15.25 ± 1.69 vs. 15.52 ± 1.71 years, 161.27 ± 7.32 vs. 162.47 ± 7.67 cm, 52.57 ± 9.02 vs. 52.97 ± 13.57 kg, and 2.60 ± 0.67 vs. 3.44 ± 1.29 members, respectively. Around 45% of participants in the intervention group and 41.66% of participants in the control group were tenth-year students. The single parent of all participants in the intervention group and 86.1% of participants in the control group was their mothers, and 40% of parents in the intervention group and 47.2% of parents in the control group had diploma. Most participants in both groups were the first child of family (75% vs. 55.55%). Moreover, most parents in the intervention group were employed (62.5%), while most parents in the control group were housewife (58.3%). Half of the participants in both groups reported insufficient monthly family income. Groups did not significantly differ from each other respecting participants’ age, height, weight, educational year, monthly family income sufficiency, and their parent’s educational level (*P* > 0.05; Table 2).

**Table 2:** Between-group characteristics with respect to participants’ demographic characteristics

Characteristics	Groups	Intervention Mean ± SD or N (%)	Control Mean ± SD or N (%)	*P* value
Age (Years)		15.25 ± 1.69	15.25 ± 1.71	0.48*
Height (Centimeters)		161/7 ± 27/32	162/47 ± 7/67	0.48*
Weight (Kilograms)		52/9 ± 57/02	52/13 ± 97/57	0.88*
Family size		2/60 ± 0/67	3/1 ± 4/29	0.001*
Educational year	Seventh	8 (20)	7 (19.44)	0.598^
	Eighth	6(15)	3(8/33)	
	Ninth	9(22/5)	7(19/44)	
	Tenth	9(22/5)	8(22/22)	
	Eleventh	1(2/5)	5(13/89)	
	Twelfth	7(17/5)	6(16/67)	
	Total	40(100)	36(100)	
Birth rank	First	30(75)	20(55/55)	0/004^
	Second	10(25)	8(22/22)	
	Third or more	0(0)	8(22/22)	
	Total	40(100)	36(100)	
Parent	Mother	40(100)	31(86/1)	0/02^
	Father	0(0)	5(13/9)	
	Total	40(100)	36(100)	
Parent’s educational level	Illiterate	4(10)	4(11/1)	0/87^
	Basic	12(30)	8(22/22)	
	Diploma	16(40)	17(47/2)	
	University	8(20)	7(19/5)	
	Total	40(100)	36(100)	
Parent’s employment status	Employed	25(62/5)	13(36/11)	0/024^
	Unemployed	0(0)	2(5/55)	
	Housewife	15(37/5)	28(58/3)	
	Total	40(100)	36(100)	
Monthly family income sufficiency	Insufficient	20(50)	18(50)	1.00**
	Relatively sufficient	12(30)	11(30/6)	
	Sufficient	8(20)	7(19/4)	
	Total	40(100)	36(100)	

*The results of the independent-sample t test

**The results of the Chi-square test

^The results of the Fisher’s exact test

There were no significant differences between the study groups respecting the pretest mean scores of HPL and its dimensions (*P* > 0.05), except for the nutrition dimension which was significantly higher in the intervention group (*P* = 0.003). After the intervention, the mean scores of HPL and its dimensions in the intervention group were significantly higher than the control group (*P* < 0.05), except for the health responsibility dimension (*P* = 0.362) (Table 3). After adjusting the effects of the significant between-group difference respecting pretest mean scores, the pretest–posttest differences of the mean scores of HPL and all its dimensions in the intervention group were significantly more than the control group (*P* < 0.05) (Table 4).

**Table 3:** Between-group comparisons respecting the pretest and the posttest mean scores of health-promoting lifestyle and its dimensions

Dimensions	Time	Before		After	
		Mean ± SD or Median (IQR)	*P* value	Mean ± SD or Median (IQR)	*P* value
	Group				
Nutrition	Intervention	11 (10, 12)	0.003*	12 (11, 13)	< 0.001*
	Control	10 (8, 11)		9 (8, 11)	
Physical activity	Intervention	10(7,12)	0/324*	12(11,15)	0/003*
	Control	12(8/25,13)		10(8,13)	
Health responsibility	Intervention	12.35 ± 3	0.134^	13.10 ± 3.66	0.362^
	Control	13.36 ± 4.44		12.27 ± 4.15	
Stress management	Intervention	11/50(10,13)	0/165*	13(11,14)	0/005*
	Control	13(10/25,14)		11/50(8/25,13)	
Social support	Intervention	13/50(10,16)	0/601*	17(15,18)	0/001*
	Control	12(9,15)		13/50(10,15)	
Life perception	Intervention	16(15,17)	0/257*	18(16,20)	0/018*
	Control	18(14,19)		16(13/25,18)	
Total	Intervention	72/80 ± 9/30	0.805^	82(78,90)	0.001*
	Control	73/58 ± 16/74		71/50(63/25,84/50)	

*IQR* Interquartile range

*The results of the Mann–Whitney *U* test

^The results of the independent-sample *t* test

**Table 4:** Between-group comparisons respecting the pretest–posttest differences of the mean scores of HPL and its dimensions

Dimensions	Median (Interquartile 1,3)		*P* value*
	Intervention Median (IQR)	Control Median (IQR)	
Nutrition	1 (0, 3)	0 (–1, 0)	0.002
Physical activity	2/50(1,4)	0(–1,0)	0.000
Health responsibility	1/50(–1,3)	0(–2,0)	0.002
Stress management	1(0,4)	0(–1,0)	0.001
Social support	2(1,4)	0(–1,1)	0.001
Life apperception	1/50(0,4)	0(–1,0)	0.001
Total	10(6,16)	–1(–5/50,1)	0.000

*IQR* Interquartile range

^*^The results of the Mann–Whitney *U* test

## Discussion

The aim of this study was to investigate the effects of VL on HPL among single-parent adolescent girls during the COVID-19 pandemic. Findings showed that VL significantly improved HPL among these girls. This is in agreement with the findings of previous studies [20, 31, 35, 38, 43–45].

The findings of the present study revealed that VL had significant positive effects on the nutritional behaviors of single-parent adolescent girls. Healthy nutrition is a key component of adolescent health [10, 46]. Nutritional behaviors are determined by many different factors, including despair, lack of motivation, stress, and concerns [47]. The VL intervention of the study might have improved the nutrition mean score through reducing participants’ despair and improving their motivation.

We also found that VL significantly improved participants’ mean score of physical activity. This dimension refers to a healthy and regular physical activity pattern in lifestyle [40]. Starting and continuing a new behavior always need motivation and perseverance [31] and hence, most successful programs on physical activity include motivational techniques [48]. Our VL intervention might also have improved physical activity among participants through improving their motivation for engagement in physical activity.

Study findings also revealed significant increase in the mean score of the health responsibility dimension of HPL after VL. This is in line with the findings of a study in China which showed that meaning-based psychological intervention significantly improved health and life responsibility in Chinese college students [49]. Learning and choosing a healthy lifestyle are among the health responsibilities of all individuals [40]. Logotherapy helps individuals consider themselves responsible towards their pain and suffering instead of considering themselves as the victims of pain and suffering [45]. Healthy individuals know that greater latitude is associated with greater responsibility and hence, feel greater responsibility towards their choices and behaviors. Greater health responsibility requires individuals to choose a healthy lifestyle and engage in activities such as healthy eating, avoidance from cigarette smoking and alcohol consumption, and immunization against diseases in order to prevent diseases and promote their health [40].

We also found that VL significantly improved participants’ stress management ability. Single-parent adolescents are vulnerable to stress. Different studies in Iran have shown that logotherapy helps individuals find meaning and purpose in life and thereby, improves their general health and quality of life and reduces their anxiety and depression [32–35, 38]. Previous studies in Iran and also a metaanalysis in London reported that logotherapy reduces stress through improving stress management ability [32, 35, 50–52]. Another study showed that a meaning-based intervention reduced stress and depression during the COVID-19 pandemic [27]. An explanation for the positive effects of logotherapy on stress is that it helps individuals use their abilities and commitment to accept difficult and stressful life events, less frequently experience frustration and disappointment in difficult conditions, find meaning in life, improve their self-esteem, and feel lower stress and anxiety [19, 36, 44, 52].

Our findings also indicated that VL had significant positive effects on the social support dimension of HPL. The social support can positively affect engagement in HPL behaviors [11, 46, 53–55]. In line with our findings, some previous studies in Iran, reported the effectiveness of logotherapy in improving interpersonal relationships and perceived support [32, 43, 45]. A study also showed that meaning-based psychological intervention helped college students appreciate their families and friends and helped them establish more relationships with them [49]. Another study in Netherlands also revealed that meaning-based group psychotherapy improved peer support and reduced loneliness [56]. Moreover, a study in Egypt reported that empowering individuals to find meaning in their life based on social relationships had positive effects on their social networks [30]. A study in Australi also reported logotherapy as an effective intervention to reduce social isolation during the COVID-19 pandemic and recommended the use of VL for well-being improvement [39]. Logotherapy helps individuals learn how to establish relationship in groups, correct their interpersonal relationships, show greater adaptation in their emotional and social relationships, and hence receive greater emotional and social support [32, 45].

VL in the present study also significantly improved life appreciation among single-parent adolescent girls. Life appreciation refers to purposefulness in life [40] and can improve physical and mental well-being through increasing motivation for behavior modification [57]. A study in Iran showed that purposeful life training can significantly improve life appreciation and help individuals move towards purposefulness in life [57]. Another study in China also showed that meaning-based intervention improved life appreciation and helped individuals find clear purposes in life [49]. Because of puberty- and identity-related crises and changes, most adolescents attempt to understand meaning and face questions such as, “Who am I?”, “What am I doing here?”, and “What has been the goal of my creation?” [45]. If they cannot find answers to such questions, they may experience despair and even feel that there will be no future for them [45]. Logotherapy can provide a conceptual framework to help individuals find meaning in life [44, 45]. It also helps individuals understand that different situations can facilitate or complicate how to find meaning in life, while meaning is always present and achievable in life even in the most adverse life conditions [36, 44].

The logotherapy intervention in the present study was virtually implemented due to COVID-19-related restrictions such as physical distancing. Most healthcare providers resorted to virtual education during the COVID-19 pandemic [24]. A study in Canada showed a significant rapid transition to virtual methods in healthcare service delivery during the first twelve months after the onset of the COVID-19 pandemic [58]. Another study in Dubai also reported the effectiveness of virtual healthcare services in fulfilling patient needs in primary care centers during the COVID-19 pandemic [59]. It seems that virtual methods in healthcare delivery will be prevalent after the COVID-19 pandemic [24] because these methods have facilitated access to healthcare services, reduced patients’ expenses, and improved healthcare providers’ relationships with their disabled or rural clients [29, 59]. Single-parent adolescents in the present study welcomed the VL intervention because they could attend VL sessions without spending any money and putting themselves at risk for COVID-19 transmission. In overall, VL intervention in the present study might have improved HPL through reducing despair and promoting hopefulness, health responsibility, and motivation for health promotion.

Some eligible girls could not participate in the study due to their limited access to smart phone. Virtual methods have some limitations such as clients’ preference to receive face-to-face services, limited access of some clients to information technology, inappropriateness of these methods for some types of counseling, lack of official guidelines, heavier workload, limited financial and organizational support, lack of the necessary infrastructures, and limited supervision [29, 59]. Effective initiatives are needed to improve telehealth infrastructures and facilitate clients’ access to virtual healthcare services.

Generally, in explaining the above results, it can be said that changing behavior, lifestyle improvement and health promotion require motivation and responsibility in people. Logotherapy is also an approach that tries to reduce frustration and create motivation in people to increase their responsibility towards their health and lifestyle. It seems that the results of this research are also due to the increase in motivation, hope, and responsibility of people under the influence of logotherapy.

### Limitations

Although participants were randomly allocated to the study groups, there were between-groups differences respecting some of the demographic characteristics of participants. Moreover, the non-normal distribution of some study variables necessitated the use of non-parametric methods for data analysis.

In addition, the inability to send a file (such as a video) with a size higher than 100 MB was one of the challenges of using WhatsApp. Also, due to the virtual nature of the intervention and the unwillingness of the participants to make a video call, it was not possible for the researcher to observe the participants and it was only possible to receive verbal and textual feedback. But the ability to send text messages on WhatsApp can be considered as an advantage. Because some participants could express their opinions more easily.

## Conclusion

VL is effective in significantly improving all aspects of HPL, namely nutrition, physical activity, life appreciation, social support, health responsibility, and stress management among single-parent adolescent girls. Therefore, healthcare authorities are recommended to use VL to promote health and prevent illnesses among vulnerable adolescents. Future studies are recommended to compare the effects of face-to-face and virtual logotherapy in different vulnerable populations such as individuals with specific diseases, addicts, and homeless individuals, as well as healthy individuals.

## Data Availability

The datasets generated and/or analysed during the current study are not publicly available due [We do not have consent from all patients to publish this data] but are available from the corresponding author on reasonable request.

## Abbreviations

VL: Logotherapy
HPL: Health-promoting lifestyle
